# Epistolary

**Published:** 1870-10

**Authors:** 


### Epistolary.

*My Dear Professor:*—An Englishman once said truthfully, but with sarcasm, that the American alone knew the use of the "small of his back," he sat upon it; and, in return, we say that if these foreigners could experience the soothing, comfortable feeling of having one's heels well elevated, the blood flowing freely from the lower extremities, thus relieving the "engorgement of gravitation," they might soon adopt the ungraceful custom, and believe that some good could come out of "Lunnen;" but, unfortunately, the national foot is too large and cumbrous to be raised above the tangent level of the floor, and they must sit, perforce, square upon their ischiatic tuberosities. Be this as it may, here we are, with our heels considerably high, looking out of our office window, enjoying the trembling sunlight, snuffing up the bracing air, watching the small, fleecy white clouds chase one another across our limited patch of blue sky, like school-boys on a romp; and thus passing the *dolce far niente* of a listless existence. And yet we are not idle, the brain is seething and writhing in the throes of composition; and the blue smoke rises and curls in fantastic wreaths and circling eddies from our pipe-bowl, consoling, to some extent, our lonesome hours, and spreading a fragrant veil above and around our head, through which we seem to see, as if in some remote land, pictures of the past,—of boyhood, of hope, of peace, and of freedom from care—gone, gone forever; pictures of the present—so gloomy, so sorrowful, and so friendless; pictures of the future—alas, the tears will well up in our eyes and obscure the view, better, perhaps, for we should seek no further.

Professor, are you a fatalist? Do you believe that, what will be will be, just as what has been *was?* Do you for a moment suppose that free agency may control and shape the destinies of men, even in the slightest degree? You have read, of course, Kant, Kane, Leibnitz, Cousin and others, and like ourselves, perhaps, gotten so thoroughly mystified by their metaphysical subtleties, as to throw them from you with disgust, and ask yourself, what was man created for? If he has volition, why do circumstances invariably control him? Ah, my friend, we may have free agency, but it is mighty limited, mighty limited, and as we crawl through this miserable existence, determining each day to follow certain rules of conduct, up rises some hydra-headed monster of circumstance to turn us into some directly opposite channel. Life is truly an enigma, and when we look around among our acquaintances, and see so many doing nothing useful, but throwing themselves away with a looseness, and fully cognizant of their own worthlessness, we can only presume them placed here as an example to better men; while again, many known to us, not a few among the profession, believe the earth turns on its axis for their special benefit, and, egotists that they are, cannot understand that if they were to "pass in their chips" to-morrow, mankind would be the gainer. It is as natural to die as to be born, so they said in the olden times, and thus, in

> "Passing thro' Nature to Eternity,
> The sense of death is most in apprehension."

But why we should be so apprehensive; why we should fear this happy transition; why *la renaissance* should present any repulsive features, we cannot comprehend, unless indeed, one may have just forfeited a large life insurance policy, or become so wedded to animal instincts and appetites, as to dread the exchange for the spiritual—dropping Swedenborg out of the question. It is a matter entirely of education. The Ancients looked upon death as a handsome young fellow, with a circlet of evergreens upon his brow, and with an inviting smile, extending a friendly hand toward us; while we present him as a tall skeleton with scythe in one hand, and an hour-glass in the other, chasing some poor fellow, with long strides and clanking joints, to his dreaded charnel house. We remember as a child, the first time having this explained to us, that for weeks after we were afraid to be alone, and could not enter a dark room without whistling our loudest and shrillest notes, the perspiration oozing from every pore, the hair rising on the back of our head, and our toes squirming and twisting, like a bunch of worms in a boy's fishing basket.

Behold the wild Indian of our own continent, taught from his infancy to believe the principle of the *lex talionis* a religion; he kills, without compunction, any who may have injured him or one of his tribe; an eye for an eye, a tooth for a tooth; it makes no difference whom he may sacrifice, but some victim must be offered to the manes of his companion. See with what stoical indifference he endures the various and devilish torments inflicted by his enemies; he gives up his life without a murmur, while chanting his death-song, and vaunting his own prowess and that of his people. He feels assured of joining the "Great Spirit," and amid the happy hunting grounds, passing an eternity of peaceful content, where the water is always sweet, and where the deer, and the buffalo, and the bear will ever be plentiful.

Again, see with what placidity the Orientalist smokes his opium, taking the crosses and burdens of life with calm philosophy, attending far more scrupulously to his religious duties than ourselves, and never fearing his future state. He feels that after "shuffling off this mortal coil" he will be translated to a Mahommedan paradise; where rivers of gold and silver flow through groves of perennial greenness; where flowers ever blossom and scent the "fainting air;" where birds of gorgeous plumage ever carol their sweet melodies; and where rosy-fingered Houris, with tinted lids and glistening skins, administer to his spiritual wants and necessities forever. What a charming philosophy with which to pass life and face a future; true, bereft of all responsibility, repressive of all energy; but as we belong to the school of Optimists, why not? When we look about us, and see mankind possessed of but *one* idea, worshiping at but *one* shrine; striving, struggling, fighting for the *one* thing, money, and, to get it, emasculating themselves of every quality of manhood; then we ask again, why not?

But to leave such reflections, and to come to more material things. In reading over the proceedings of the National Medical Association, lately assembled in Washington, we cannot for the life of us see anything to commend. How is it with so many good men together, that so little was done to advance science, to uphold the profession in this country, and to foster and enlarge the proper *esprit du corps* among us?

Instead, we find nothing but contention and strife, and every thing done to throw discredit upon us, and above all, to our utter astonishment, the appointment of the "cold water philosopher" of this city, as President of the Committee on the Code of Ethics. Can you tell us by what jugglery this was brought about? Was there no other physician from Chicago, knowing his record, to object to this outrage? If the profession at large is not aware of it, it is a well-established fact here, that he is the most open and persistent violator of every principle and rule contained in that code, and, except among the small circle of parasites and flatterers, who feed upon the crumbs thrown them, there exists positively none but who complain, and will testify to his ignoble and unprofessional conduct. It has been so with him for years, and this " peripatetic professor " stalks unblushingly abroad, to injure and defame every physician whom the chances of practice unfortunately bring him in contact with. So far has this evil reached, that in the neighboring towns and cities many practitioners refuse absolutely to consult with him; and in this city, we are acquainted with numbers of excellent men, who would throw up their cases before permitting him to cross the threshold, while they were in attendance. It is well understood, that it is his invariable rule, either directly or by inuendo, to weaken the attending physician, instead of instructing, or strengthening his position; and if he can do it in no other way, by charging such small fees—though he is the loudest declaimer for a high tariff—as to appeal to the weakest side of human nature. As for ourselves, we do not know him personally, but we have always refused to meet him, and shall do so to the end of time, not only on this account, but we have little faith in the man of hobbies, whose therapeutical combinations—anywhere from 4 to 40, good for the druggist if not for the patient—imply obscure diagnosis, and seem framed upon the principle, that if one remedy won't avail, a dozen or more may hit the mark.

And how, or by what right does the " ubiquitous doctor " represent in every medical assemblage the profession of this city? We have here many men, well educated, of ripe experience, and of sound judgment, far abler to practice consistently and conscientiously, who have no say in this matter, as they are not members of his *clique*, nor belong to the Chicago Medical Society. It is about time that such outrages be checked, and we invite all members to forward their complaints, properly substantiated, that we may embody them in a protest to be presented to the National Association, to meet next year in San Francisco. It shall be our duty to see if stubborn facts can be controverted, and if such backsliding may continue with impunity, or without exposure.

And now to finish, let us say, *place aux dames*, and quote from an old author:

> " La femme souvent varie,
> Bel fol est, qui s'y fie."
>
> A woman so often varies,
> That he who trusts has *rabies*.
> (Free translation *after* Hood.)

We take the above as the text, and you will permit the introduction of an episode, a romance in real life to a certain degree, which, as our JOURNAL lays some claim to literary merit, may not be out of place. Several months back, we were called upon to attend a young gentleman in the last stages of consumption, and though his physical decay was too far advanced to hold out the least shadow of hope, yet we were soon convinced that there existed some mental element, in all probability lighting up the disease *ab initiô*, and now tending to hasten its termination. After a few weeks he took us partly in his confidence, and on the night he died, while holding his poor attenuated hand, and wiping the damp of death from his white, smooth forehead, he drew us gently towards him, and placing a letter in our hands, said, "Doctor, when I am gone, think kindly of me, do not suppose me too weak and foolish, because I have repeated the history of the world; a history beginning with Adam; has been for six thousand years, and will be for six thousand more, should the world last. I loved, I trusted, and I was deceived. The paper you hold, will give the few links wanting, and you will then the better understand me. If in your power ——" here the voice grew so feeble, that with our ear to his mouth we could barely catch his words, while the arm clasped around our neck began to relax, "——follow her up and see if——she cannot be turned——from such a course——" These were his last words, he grew weaker and weaker, and in the long solemn hour after the midnight, his soul, with many mutterings to her he loved, went out through the valley of the shadow of death, to his Maker.

C——, July, 186 .

For the love I bore you, I labored earnestly and faithfully many years, to keep you pure and undefiled; but, alas, you *would* not understand me, and you *dared* not appreciate my efforts in your behalf, for they would militate against your natural instincts, and would keep you out of that "giddy whirlpool" of meretricious display and fashion, termed the world. Go on; time will come, too late for my happiness, too late for your salvation, but it will come with inexorable certainty, when in an agony of spirit, you will exclaim on your bended knees, Oh, God! *he loved me truly*, and had I heeded his admonitions, all would have been well. I have not studied you in vain for so many years. I *do* know you, and I *do* understand the demands of your nature. No one man, were he an Apollo in beauty, a Pericles in wisdom, or an Alexander in manly enterprise, would content you, or allay those feverish longings for worldly dress and ornamentation; aye, even to the sacrifice of every quality which should ennoble the name of woman. Do you ever think of your childhood? and the tender embraces of your mother? And does not the shadow of her purity and virtue ever fall upon you? Have you forgotten the endearments of your kind father? And do not such reminiscences beckon you back from the road to sin and crime upon which you entered in ——, came here to follow, and been longing for—though held back by a loving hand—since your advent in this city? Oh! I much fear such qualms do not often come to you, for now you seem to have truth for none; your sincerity a deception; your professed love a sham; and your heart—if you ever possessed such a thing—can be made to flutter by the presence or attractions of any man, be he patrician, or be he proletarian. Though I began slowly to recognize such traits in your character, still I loved you to adoration, and had almost foresworn my God for your sake; but, as the truth forced itself upon me, the little manhood and self-respect left, arose in my defense, and declared me no longer a slave to be trampled upon, and death before such dishonor. Being ever a free-thinker, that is, holding my own opinions on many subjects apart from the world around me, I did, and do now, believe that leniency will be shown those committing many of the so-called sins, springing from tenderness of feeling and from the heart; and for all that has passed between us, or rather for the part I took in this unfortunate and wretched *liaison*, I am ready and willing to be arraigned, and feel assured that there will be forbearance and mercy for my many sufferings; as this love, so new, so fresh, so pure, elevated my life, rendered me truer to my manhood, sublimated every noble feeling of my nature, and was the means of turning me from many evil courses, incidental to the life of every man. You went to D—— twice, since I have known you, ostensibly for business purposes, and there committed with a low man, such crimes as to stain your reputation, and reeking from this bed of shame, you returned here and professed to love *me*. This I did not find out until your own conduct aroused my suspicions, but I did find out *all*, and substantiated afterwards by your voluntary confession, in which you simply declared, "because he was so kind to me;" without however, one iota of affection to excuse you, and without one word of the small sum of money received for this barter of your womanhood and honor. In my infatuation I forgave even this, hoping that with me, and under my advice, guidance and assistance, you would endeavor to lead a better life, but I found the seeds of impurity in your blood, which must blossom into crime, or you would forego your nature. I am wearied almost to the death. I see that the struggle made so desperately has been useless, and as my future was staked upon this issue, I can only add, that he who puts his trust in woman had better rest in his grave. Do not imagine for a moment, that because a few disreputable women manage to lead the life of a gaudy butterfly, accumulate a little property (the wages of sin), and occupy a *quasi* respectable position in society, that all thus succeed. Far from it; the vast majority associate only with a questionable class of men and women, without love or friendship, and before long the inevitable laws of fate revenge their outraged femininity, and hasten them to untimely and dishonorable deaths. And as a last appeal, let me beg you to desist; let me implore you by the memory of all that was ever dear to you, to turn before it may be too late. Struggle on, no matter how great the deprivations, how many the burdens, but struggle on; and, with a watchful Providence to assist you, you may yet be saved. I say for the last time, farewell; we can never meet again; the touch of the destroying Angel is on me, and before many months my troubled life will have closed. I do not despise you, though you have wrecked me, but I do pity you from the bottom of a poor crushed heart. I shall pray for you far more earnestly than for myself, and if in the coming life it be granted to watch and guide those on earth, I shall be with and protect you.

Farewell—Farewell.

Yours, Sincerely, D———.

This letter was evidently written under great mental distress, and the strength for such a task deficient; besides, many words, almost sentences obscured by the "dropping tears" of my poor patient. We have corrected such defects as lay in our power, and present it, as the history of a sad, a true, and a tender love, that which could emanate only from one of the most generous impulses, and would have rendered any true woman's life supremely happy. What must have been the feelings of that miserable creature, after reading such an appeal from a dying lover? We have seen her since, and the furrows and lines of sorrow, but a desperate resolve, are plainly marked on her sweet face. She seeks no palliation except in excess, and crime brings no blush to her cheek.

Poor soiled dove; thrown out of the pale of society by your own acts, man might forgive, and the Young Men's Christian Association might save; but your own sex, with the implacable animosity of the Furies, will pursue and hunt you to your death.

VIATOR.

CHICAGO, Sept., 1870.

